# Construction of a Bayesian network-based risk prediction model for hepatocellular carcinoma in cirrhotic patients

**DOI:** 10.3389/fonc.2025.1735042

**Published:** 2026-01-12

**Authors:** Ni Ma, Jingwei Song, Yuqing Yang

**Affiliations:** 1 School of Public Health, Xinjiang Medical University, Urumqi, China; 2 The Second Affiliated Hospital of Xi’an Jiaotong University Xinjiang Hospital, People’s Hospital of Xinjiang Uygur Autonomous Region Bainiaohu Hospital, Urumqi, China

**Keywords:** Bayesian network mode, cirrhosis, hepatocellular carcinoma, influencing factors, risk prediction

## Abstract

**Objective:**

To investigate clinical data of hospitalised cirrhosis patients, identify risk factors for cirrhosis progression to hepatocellular carcinoma, establish a risk prediction model, and provide scientific basis for early identification of high-risk patients.

**Methods:**

Hospitalised cirrhosis patients treated at Xinjiang Uygur Autonomous Region People’s Hospital between January 2019 and December 2023 were selected. Their inpatient examination records were retrieved, including medical record summaries alongside results for coagulation function, complete blood count, liver function tests, urinalysis, renal function tests, tumour markers, comprehensive hepatitis panel, comprehensive thyroid function panel, comprehensive biochemical panel, glucose series, and lipid series. Patients diagnosed with hepatic malignancy during subsequent hospitalisations (excluding the initial admission) formed the cancer progression group, while those without hepatic malignancy constituted the control group. Univariate and multivariate analyses identified risk factors for hepatocellular carcinoma (HCC) progression in cirrhosis patients. A predictive model for HCC development in cirrhosis patients was constructed using a combined Lasso regression model and Bayesian network model.

**Results:**

This study enrolled 1,204 individuals, including 1,128 cirrhosis patients, of whom 76 progressed to liver malignancy. Multivariate logistic regression analysis indicated that female gender was a protective factor against cirrhosis progression to liver malignancy(*OR* = 0.532, *OR*95% *CI* = 0.297-0.952); while hepatitis B, elevated total cholesterol, and reduced antithrombin III activity were risk factors for progression to hepatocellular carcinoma (*OR* = 4.080, *OR*95%*CI* = 2.443-6.814;*OR* = 2.308, *OR*95%*CI* = 1.132-4.707, *OR* = 2.982, *OR* 95% *CI* = 1.389-6.402) (*P* < 0.05); The LASSO regression model ultimately identified 14 variables most significantly associated with the transformation of cirrhosis into malignant liver tumours: PT, TT, MAO, ALP, PDW, CRP, CK-MB, Ca, P, TC, AFP, FT4, SCC, AT3. The results of the Bayesian network model indicate that DOI, AT3, SCC, CRP, and TC have a direct connection with the occurrence of hepatic malignancy; Gender, FT4, hepatitis type, and Ca indirectly influence the development of hepatic malignancy. Using sensitivity analysis, we examined the probability of cirrhosis patients progressing to hepatocellular carcinoma under specific conditions. The results indicated that the probability of liver cirrhosis patients progressing to hepatocellular carcinoma was 0.0749 (7.5%) when AT3 levels were normal, 0.0709 (7.1%) when AT3 was low, and 0.0850 (8.5%) when AT3 was high. When TC levels were normal, the probability was 0.0763 (7.6%), and it increased to 0.1309 (13.1%) when TC was elevated. The performance of the Bayesian network model (AUC = 0.857, Brier Score = 0.052, Accuracy = 0.940, C-index = 0.833 (95% *CI*: 0.784–0.882), Sensitivity = 0.836, Specificity=0.744) was superior to that of the logistic regression model (AUC = 0.780, Brier Score = 0.056, Accuracy = 0.934, C-index = 0.785 (95% *CI*: 0.726–0.843), Sensitivity = 0.727, Specificity = 0.766). Ten-fold cross-validation showed an average accuracy of 0.93. After balancing sensitivity and specificity, the optimal threshold was determined by maximizing the Youden index (0.052), with a predicted probability >0.052 indicating progression from liver cirrhosis to hepatic malignancy. Based on the ROC curve, threshold 1 was set at 0.2 and threshold 2 at 0.8, establishing risk stratification as follows: low risk (predicted probability <0.2), intermediate risk (0.2 ≤ predicted probability ≤ 0.8), and high risk (predicted probability >0.8). This resulted in 318 patients classified as low risk, 42 as intermediate risk, and 1 as high risk.

**Conclusions:**

Gender, hepatitis B, TC, and AT3 constitute risk factors for hepatocellular carcinoma in cirrhotic patients; Gender, hepatitis type, DOI, FT4, AT3, SCC, CRP, MAO, and Ca are associated with the progression of liver cirrhosis to malignant liver tumours either directly or indirectly. The risk prediction model constructed by combining LASSO regression with Bayesian networks demonstrates good predictive value.

## 1 Introduction

Liver cancer, specifically hepatocellular carcinoma (HCC), is a malignant tumour originating from liver cells, primarily comprising two types: hepatocellular carcinoma and cholangiocarcinoma. It ranks among the most prevalent malignant tumours globally, exhibiting high incidence and mortality rates. According to 2020 estimates, hepatocellular carcinoma ranks sixth among the most common malignant tumours worldwide, third among causes of malignant tumour mortality (1), and second among causes of premature cancer death (2). Hepatocellular carcinoma presents insidiously, with a poor prognosis in advanced stages, a median survival of less than one year, and a five-year survival rate of merely 7% (3, 4). Substantial evidence indicates that cirrhosis constitutes the primary pathological basis for the development of hepatocellular carcinoma (HCC), with over 80% of HCC cases originating from this population (5). Consequently, it has become an international consensus to identify cirrhosis patients as the core target group for primary prevention and early monitoring of HCC. Current clinical practice guidelines recommend regular ultrasound screening for all patients with liver cirrhosis. However, significant heterogeneity exists within this cohort, and the risk of progression to hepatocellular carcinoma (HCC) is not uniform. Therefore, precise risk stratification of cirrhosis patients to identify a truly “extremely high-risk” subgroup and develop individualised enhanced monitoring protocols for them is crucial for optimising HCC prevention and control systems and improving patient outcomes. This study aims to identify risk factors for the progression of liver cirrhosis to hepatocellular carcinoma by analysing diagnostic data from hospitalised cirrhosis patients, and to establish a predictive risk model. This endeavour seeks to provide scientific evidence for the early identification of high-risk individuals for hepatocellular carcinoma among cirrhosis patients.

## 2 Research content and methods

### 2.1 Research subjects

Patients hospitalised for liver cirrhosis at the Xinjiang Uygur Autonomous Region People’s Hospital between January 2019 and December 2023 were selected. Exclusion criteria were: ① Patients hospitalised for less than 24 hours; ② Patients with newly diagnosed malignant liver tumours during their first admission; ③ Patients with secondary liver cancer; ④ Patients with concomitant other tumours.

### 2.2 Research content

An analysis of the general characteristics and examination results of patients with cirrhosis and those who progressed from cirrhosis to hepatocellular carcinoma was conducted. This identified the factors influencing the development of hepatocellular carcinoma in cirrhosis patients and enabled the construction of a risk prediction model for the progression from cirrhosis to hepatocellular carcinoma.

### 2.3 Research methodology

The hospital information department retrieved medical records for cirrhosis patients using their hospital admission numbers as identifiers. This included the front pages of medical records and test results for coagulation function, complete blood count, liver function tests, urinalysis, renal function tests, tumour markers, comprehensive hepatitis panel, comprehensive thyroid function panel, comprehensive biochemical panel, glucose series, and lipid series. A total of 97 variables were selected, with incomplete or missing data removed from the records.

Patients with liver cirrhosis who were diagnosed with hepatic malignancy during subsequent hospitalisations (excluding the initial admission) constituted the malignancy group, while those without hepatic malignancy served as the control group. The progression of liver cirrhosis to hepatic malignancy was confirmed through clinical presentation, imaging studies, and pathological diagnosis. This study was approved by the hospital’s ethics committee (KY2024052420).

Diagnostic criteria for malignant liver tumours: Guidelines for the Diagnosis and Treatment of Hepatocellular Carcinoma (2019 Edition) (6).

Diagnostic criteria for cirrhosis: Evidence-based clinical practice guidelines for Liver Cirrhosis (7).

### 2.4 Statistical methods

1. Use Excel software to perform preliminary data processing.
2. Univariate analysis and factor analysis were conducted using SPSS 26.0 software. Numerical variables were expressed as mean ± standard deviation, with intergroup comparisons performed using the independent samples t-test. Categorical variables were represented by frequency and proportion, with intergroup comparisons conducted using the chi-square test. Factor analysis employed a binary logistic regression model. The significance level was set at *P* < 0.05.
3. Handling Missing Values and Outliers: Missing values were detected using the summary function in R. If any missing values were found, the Multiple Imputation method was employed for their imputation. Outliers were detected using boxplots, where values below Q1-1.5 * IQR or above Q3 + 1.5 * IQR were considered outliers. If an outlier resulted from an operational error during data recording, the data were re-verified and corrected. If the outlier was a natural component of the population, the winsorization method was applied to adjust the outlier to the boundary value of the corresponding percentile.
4. Employing the glmnet package within R software for LASSO regression variable selection, cross-validation was utilised to identify the optimal λ. Model λ screening primarily encompasses lambda.min and lambda.1se; this study employed lambda.min to select variables most strongly associated with the outcome.
5. Bayesian network modelling was conducted using the bnleam package within R software. Divide the data into training and test sets in a 7:3 ratio. First, use the training set data with the hc function to train the network, obtaining a preliminary network architecture. The bn.fit_MLE function was then used to learn the network parameters. The cpquery function facilitated Bayesian network inference and the calculation of conditional probability distribution tables. Subsequently, nodes and directed links may be added to the Bayesian network model based on existing research findings and decision tree models, employing the set.arc function for this purpose. Finally, Netica software was used to construct the Bayesian network model. The built-in node inference function of Netica was employed to calculate the probability of outcome changes caused by the cumulative effects of various factors. Additionally, sensitivity analysis was conducted using the “grain”package in R to analyse the probability of the outcome under specific single-factor conditions.
6. Model Evaluation: The “ggplot2” package in R was used to plot calibration curves. By comparing the Bayesian network model with the logistic regression model, the predictive performance of the Bayesian network model was assessed through a comprehensive comparison of several evaluation metrics, including Brier Score, AUC, Accuracy, C-index (95% *CI*), Sensitivity, and Specificity. k−fold cross-validation was employed to evaluate the accuracy rate of the model.
7. Model Prediction Threshold and Risk Stratification Strategy: The trained Bayesian network model was applied to the test set to compute risk probabilities. After evaluating the model’s performance, the ROC curve and AUC were calculated. The optimal threshold was determined from the ROC curve by maximizing the Youden index. Based on the predicted probabilities and actual classifications, two thresholds were defined: Threshold 1 (the upper limit for low risk—the threshold at which specificity reaches 90%) and Threshold 2 (the lower limit for high risk—the threshold at which sensitivity reaches 90%). The curve was then segmented into three parts corresponding to low, intermediate, and high risk. Finally, the relationships between risk strata and predicted probabilities, risk strata and actual cancer status, as well as a histogram of predicted probability distribution, were plotted.

## 3 Result

### 3.1 General overview

A total of 1,204 individuals were included in this study, comprising 1,128 patients with liver cirrhosis, with no missing values identified. Among them, 76 patients with liver cirrhosis developed hepatic malignancy. There were 724 males and 480 females. The proportion of males progressing to liver malignancy (8.3%) was higher than that of females (3.3%). Patients with cirrhosis and hepatitis B showed the highest incidence of liver malignancy progression (13.3%). Patients with a disease duration of ≥50 years had a higher incidence of liver malignancy progression than those with other durations. Patients with normal BMI exhibited the highest incidence of liver malignancy (8.3%); cirrhosis patients with elevated total cholesterol levels demonstrated a higher incidence of liver malignancy (11.8%); cirrhosis patients with reduced antithrombin III activity showed a higher incidence of liver malignancy (8.6%) (*P* < 0.05), as shown in **Table 1**.

**Table 1:** Univariate analysis of hepatocellular carcinoma development in patients with liver cirrhosis.

Variable	Levels	Cirrhosis of the liver N(%)	Malignant liver tumour N(%)	*p*
Gender	male	664 (91.7)	60 (8.3)	<0.001
	female	464 (96.7)	16 (3.3)	
Types of hepatitis	Normal	728 (96.6)	26 (3.4)	<0.001
	Hepatitis A	2 (100)	0 (0)	
	Hepatitis B	301 (86.7)	46 (13.3)	
	Hepatitis C	84 (95.5)	4 (4.5)	
	Alcoholic hepatitis	1 (100)	0 (0)	
	Autoimmune hepatitis	12 (100)	0 (0)	
Course of the disease	0	728 (96.6)	26 (3.4)	<0.001
	<1 year	23 (95.8)	1 (4.2)	
	≥1 year and <10 years	177 (88.5)	23 (11.5)	
	≥10 years and <20 years	57 (90.5)	6 (9.5)	
	≥20 years and <30 years	71 (86.6)	11 (13.4)	
	≥30 years and <40 years	44 86.3)	7 (13.7)	
	≥40 years and <50 years	23 (95.8)	1 (4.2)	
	≥50 years	5 (83.3)	1 (16.7)	
BMI	Normal	495 (91.7)	45 (8.3)	0.037
	underweight	126 (96.9)	4 (3.1)	
	overweight	363 (94.3)	22 (5.7)	
	obesity	144 (96.6)	5 (3.4)	
Total cholesterol	Normal	1046 (94.1)	65 (5.9)	0.040
	slightly elevated	82 (88.2)	11 (11.8)	
Antithrombin III	Normal	187 (97.9)	4 (2.1)	<0.001
	below average	682 (91.4)	64 (8.6)	
	slightly elevated	259 (97.0)	8 (3.0)	

### 3.2 Risk factor analysis

The results of the multivariable logistic regression model indicate that being female is a protective factor against the progression of liver cirrhosis to hepatocellular carcinoma (*OR* = 0.532, *OR* 95% *CI* = 0.297-0.952); Hepatitis B, elevated total cholesterol, and reduced antithrombin III activity were identified as risk factors for the progression of cirrhosis to malignant liver tumours (*OR* = 4.080, *OR* 95% *CI* = 2.443–6.814, *OR* = 2.308, *OR* 95% *CI* = 1.132–4.707, *OR* = 2.982, *OR* 95% *CI* = 1.389–6.402) (*P* < 0.05), as shown in **Table 2**.

**Table 2:** Analysis of risk factors for hepatocellular carcinoma in patients with liver cirrhosis.

Variable	*β*	*Wald*	*P*	*OR*	*95% confidence interval for the OR*	
					lower limit	upper limit
Gender(female)	-0.632	4.510	0.034	0.532	0.297	0.952
Types of hepatitis		29.889	0.000			
Hepatitis A	-17.757	0.000	0.999	0.000	0.000	0.000
Hepatitis B	1.406	28.891	0.000	4.080	2.443	6.814
Hepatitis C	0.304	0.300	0.584	1.355	0.457	4.021
Alcoholic hepatitis	-18.326	0.000	1.000	0.000	0.000	0.000
Autoimmune hepatitis	-17.507	0.000	0.999	0.000	0.000	0.000
Elevated total cholesterol	0.836	5.289	0.021	2.308	1.132	4.707
Antithrombin III		15.422	0.000			
Slightly elevated	-0.489	0.608	0.436	0.613	0.179	2.098
Below average	1.093	7.859	0.005	2.982	1.389	6.402
Constant	-3.338	34.537	0.000	0.035		

### 3.3 LASSO regression model

**Figure 1:**
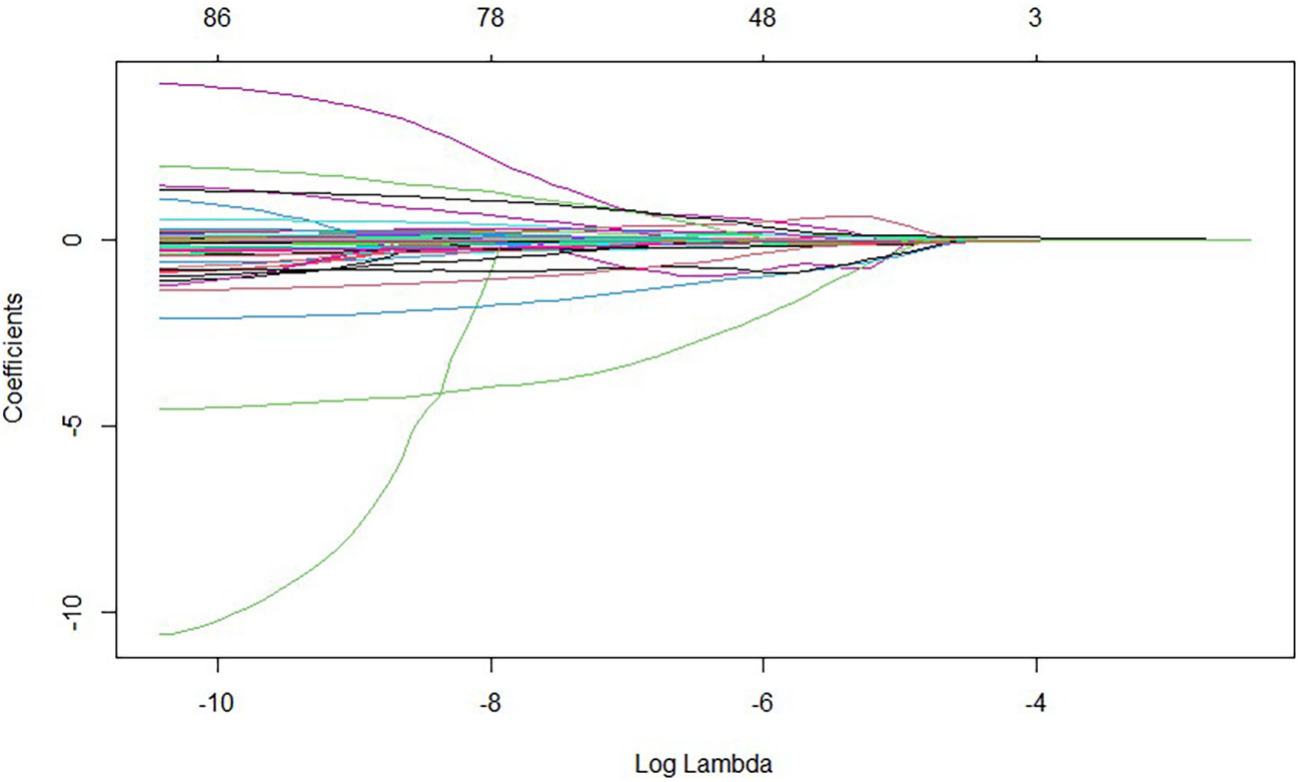
Variable selection in the LASSO-based predictive model 1.

**Figure 2:**
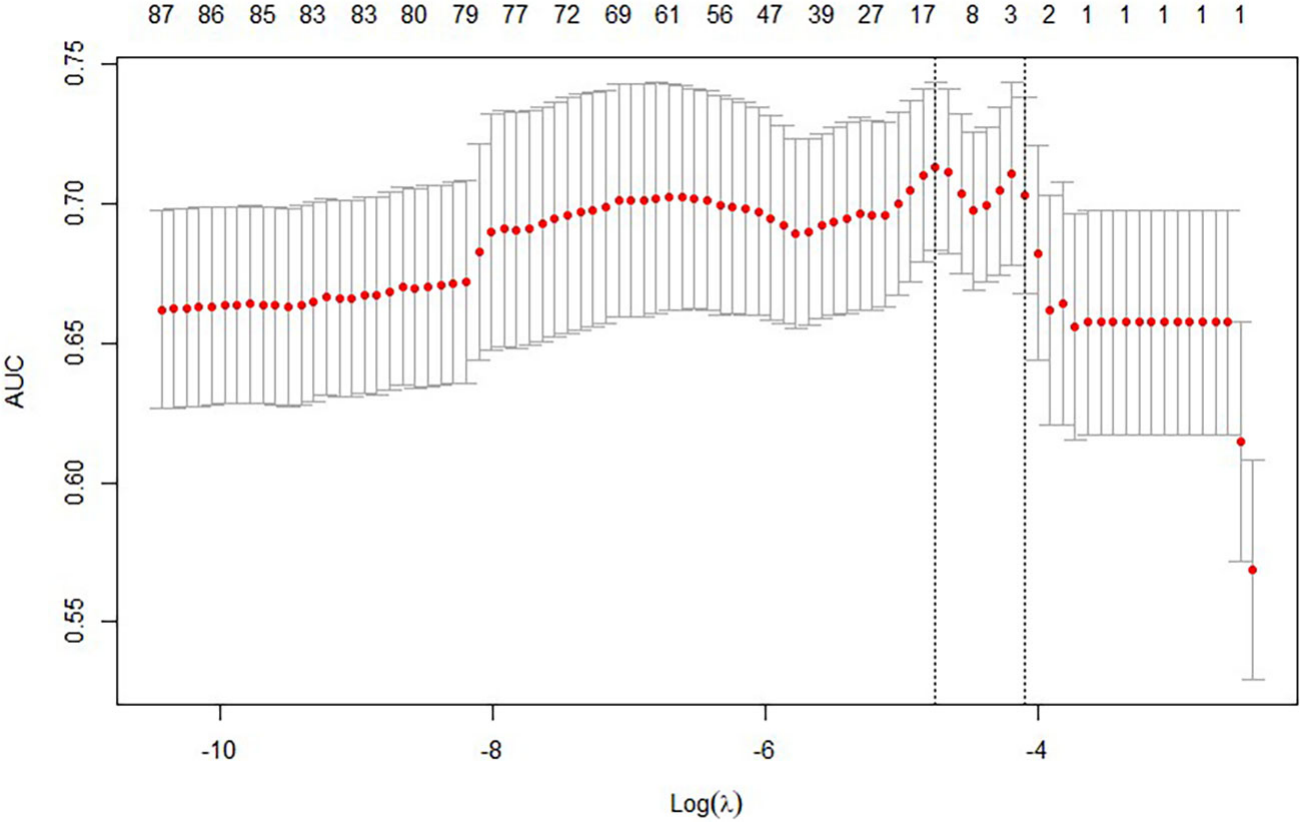
Variable selection in the LASSO-based predictive model 2.

The 97 collected laboratory test results were incorporated into the LASSO model. After 100 rounds of cross-validation, the minimum average error lambda value was determined as lambda.min=0.008638309. This lambda value was then applied to the LASSO model, ultimately identifying 14 variables most significantly associated with the progression from liver cirrhosis to malignant liver tumours. These variables are: monoamine oxidase, alkaline phosphatase, platelet distribution width, C-reactive protein, creatine kinase isoenzymes, total calcium, phosphorus, total cholesterol, alpha-fetoprotein, free thyroxine, squamous cell carcinoma-associated antigen, and antithrombin III activity. See **Figures 1**, **2**, and **Table 3**.

**Table 3:** Coefficients and odds ratios for variables selected in the LASSO model prediction.

Variable	Coefficient	*OR*
Prothrombin time(PT)	-3.66E-02	0.9640603
Thrombin time(TT)	7.75E-02	1.080562
Monoamine oxidase(MAO)	-8.12E-02	0.921985
Alkaline phosphatase(ALP)	9.77E-05	1.0000977
Platelet distribution width(PDW)	-1.06E-03	0.9989404
C-reactive protein(CRP)	3.26E-03	1.0032662
Creatine kinase isoenzymes(CK-MB)	2.31E-03	1.0023101
Ca	1.80E-01	1.1966451
P	-2.21E-01	0.8014812
Total cholesterol(TC)	3.42E-02	1.0347541
Alpha-fetoprotein(AFP)	2.79E-05	1.0000279
Free thyroxine(FT4)	-1.71E-01	0.8427542
Squamous Cell Carcinoma-Associated Antigen(SCC)	-6.85E-02	0.9337824
Antithrombin III(AT III)	1.09E-01	1.1155666

### 3.4 Construction of Bayesian network models

**Figure 3:**
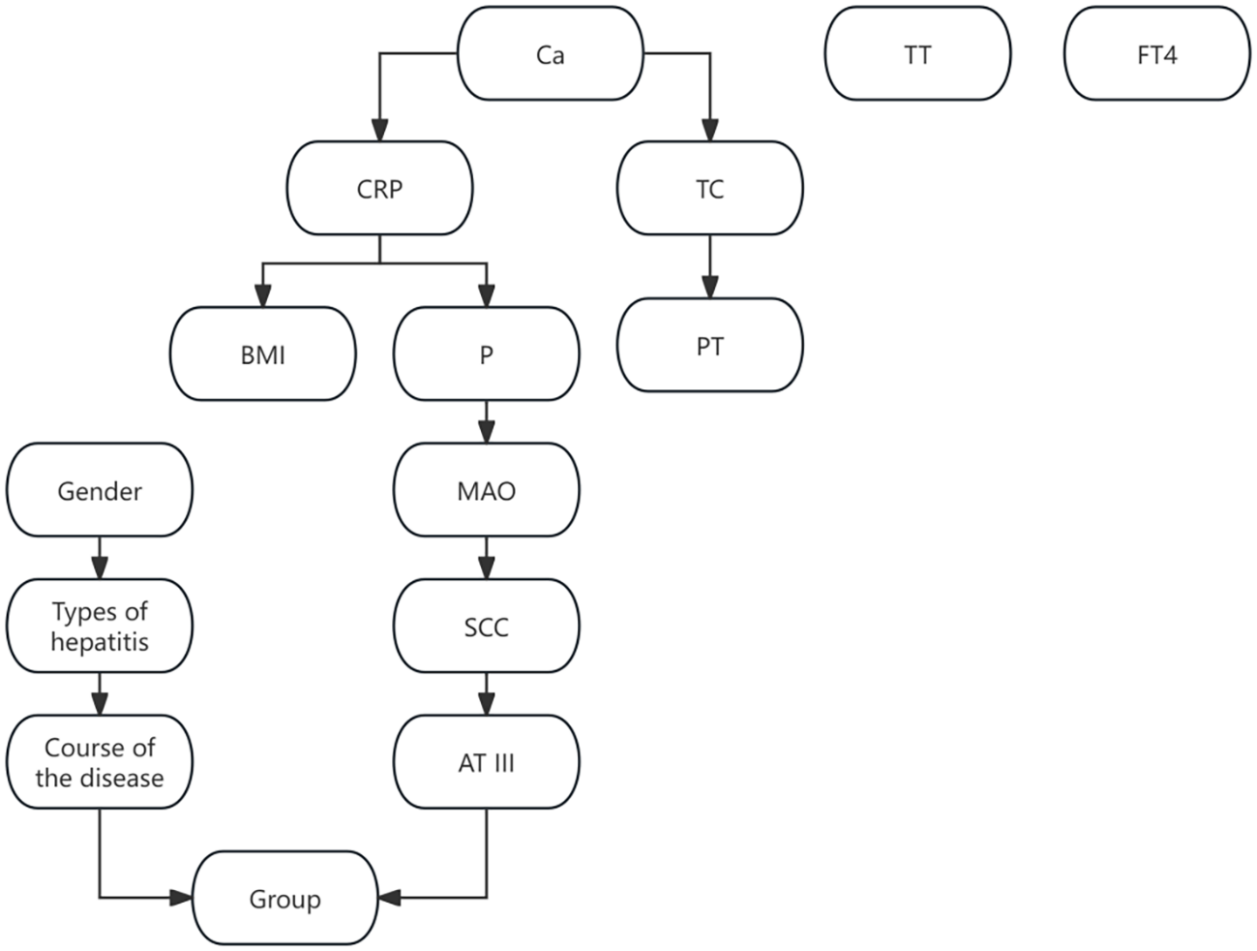
Bayesian network model.

**Figure 4:**
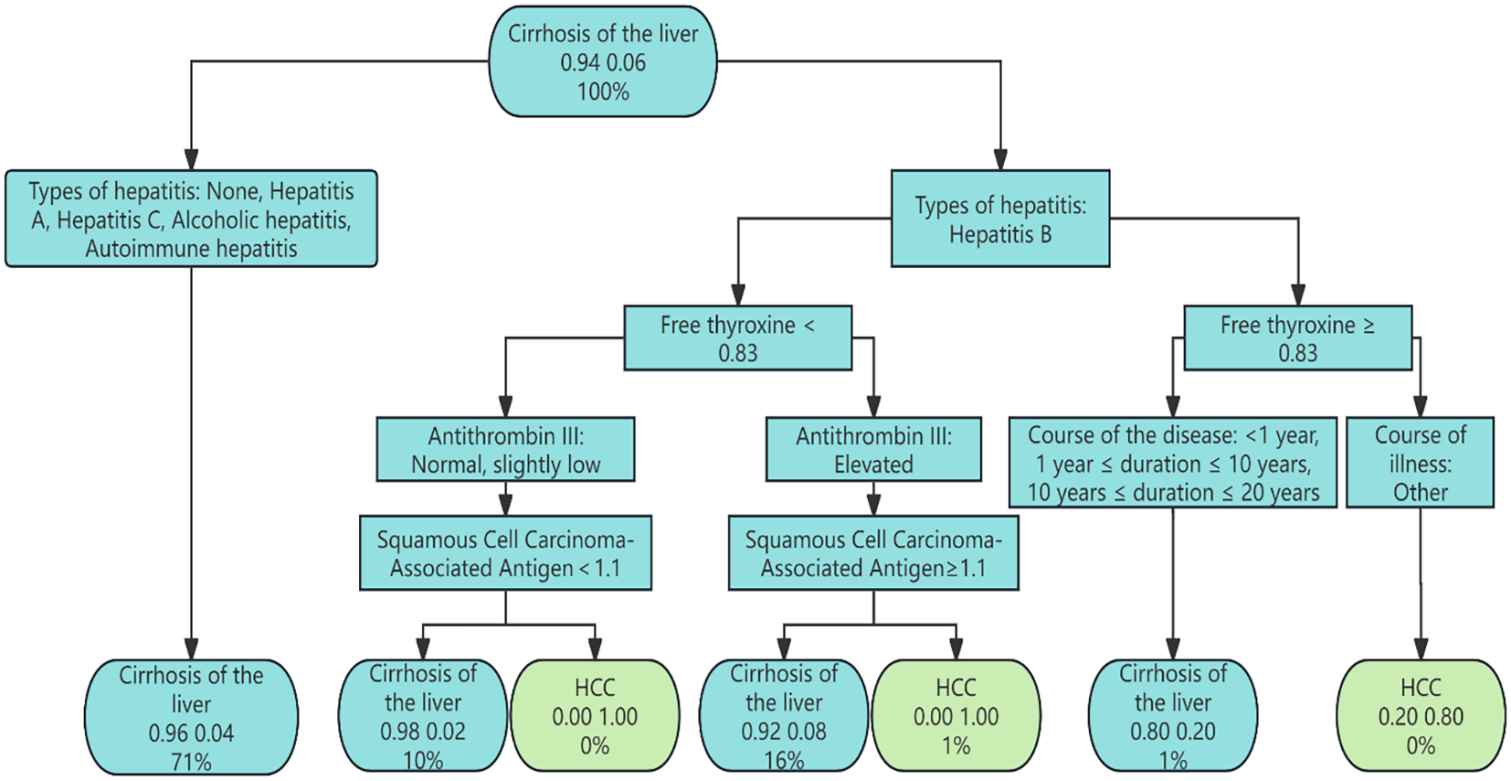
Decision tree model.

**Figure 5:**
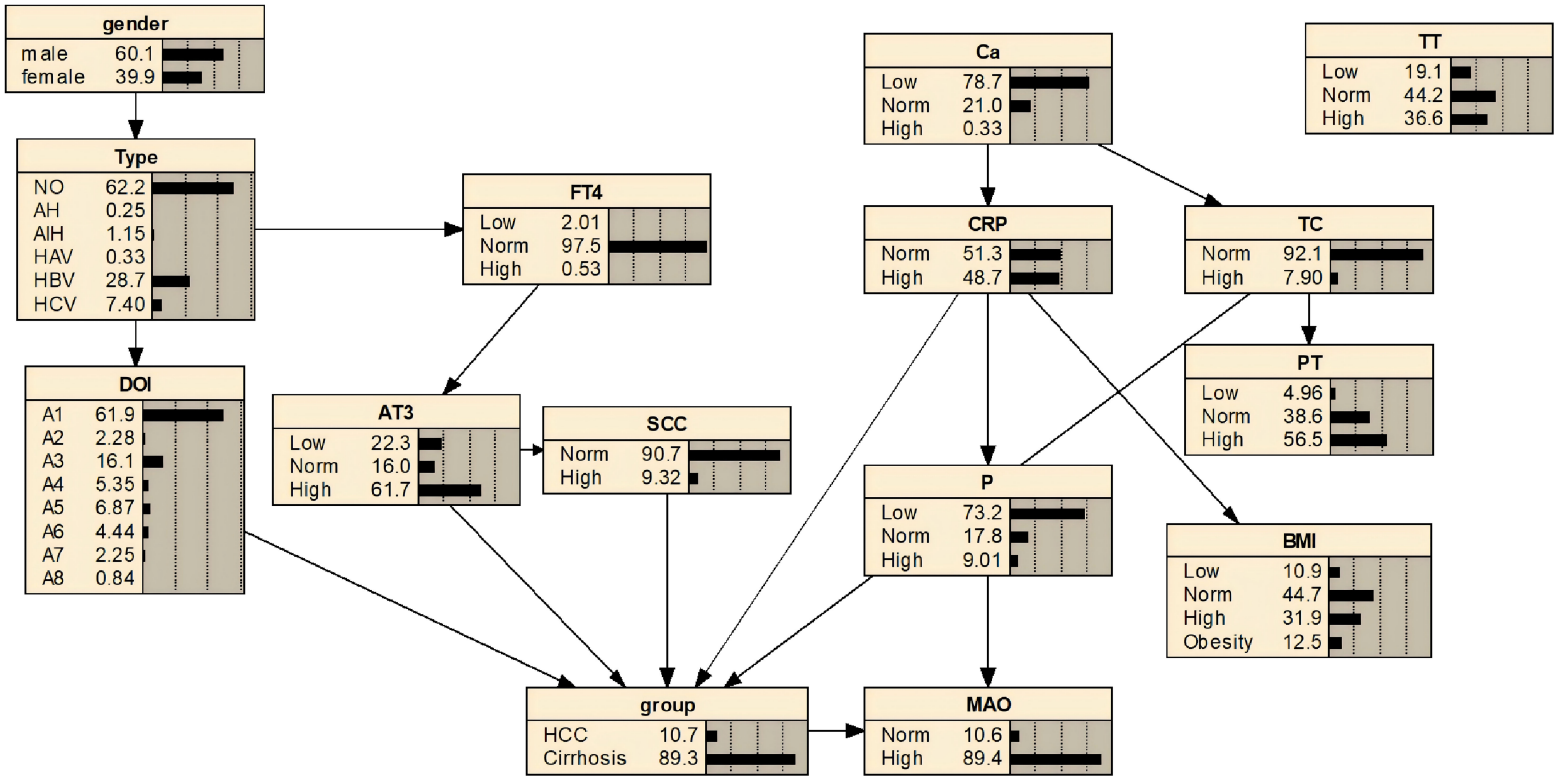
Bayesian network risk prediction model for hepatocellular carcinoma in patients with liver cirrhosis.

Based on variables selected via LASSO regression and combined with risk factor analysis results, an initial Bayesian network disease prediction model comprising 15 nodes and 12 directed edges was constructed. The conditional probabilities for each node are shown in **Figure 3**. By constructing a decision tree model, relationships among some variables were identified (see **Figure 4**). Integrating this with the Bayesian network model from **Figure 3**, the set.arc function was employed to add edges, yielding a Bayesian network model comprising 15 nodes and 17 directed edges (see **Figure 5**). **Figure 5** demonstrates that variables including gender, hepatitis type, disease duration, free thyroxine, antithrombin III activity, squamous cell carcinoma-associated antigen, C-reactive protein, monoamine oxidase, and total calcium establish connections with the progression of liver cirrhosis to malignant liver tumours through complex network relationships. Disease duration, antithrombin III activity, squamous cell carcinoma-associated antigen, C-reactive protein, and total cholesterol are directly associated with the occurrence of hepatic malignancy; gender, free thyroxine, hepatitis type, and total calcium exert an indirect influence on the development of hepatic malignancy.

### 3.5 Model inference

**Figure 6:**
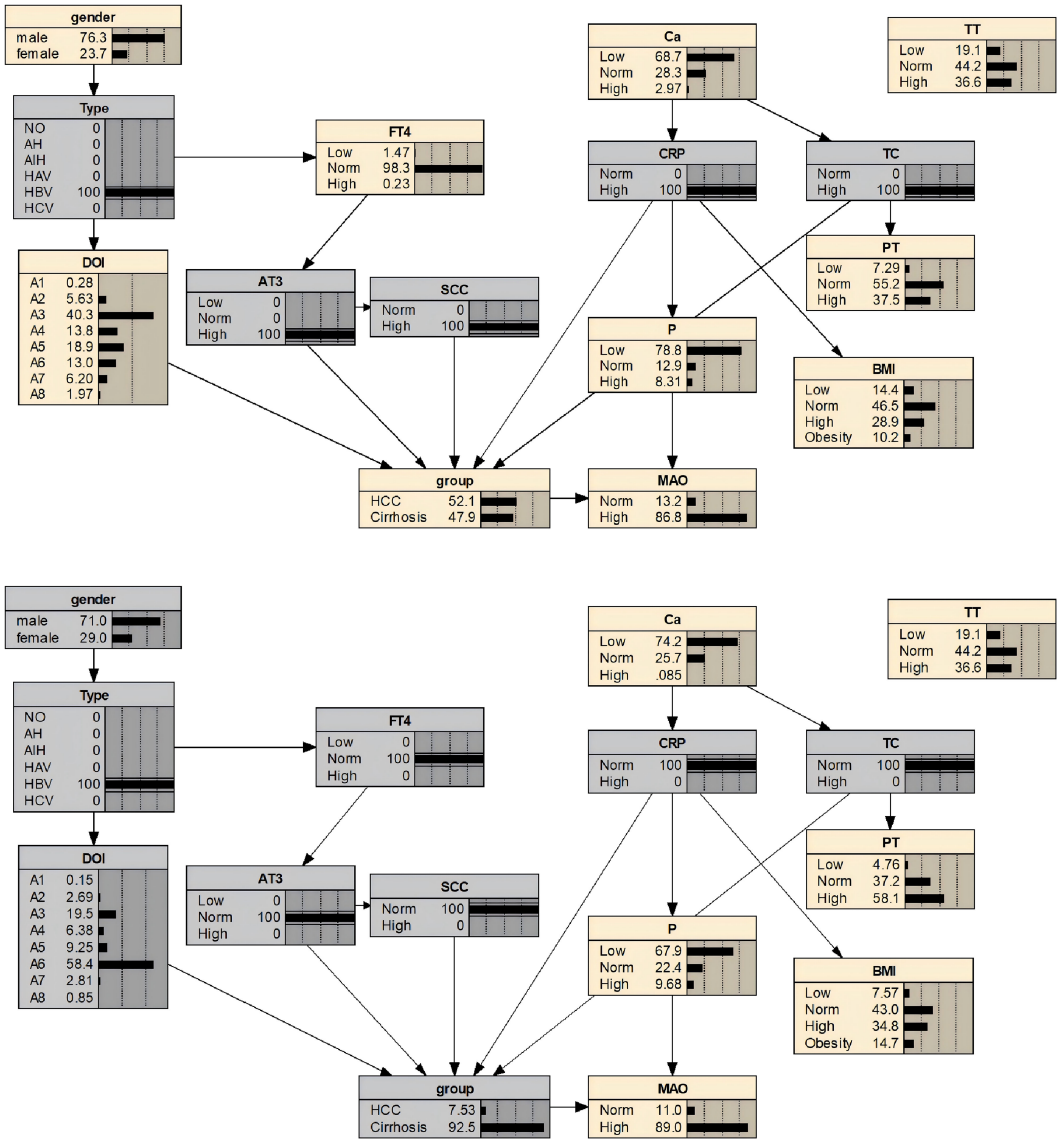
Bayesian network prediction model inference for hepatocellular carcinoma development in patients with liver cirrhosis.

Sensitivity analysis revealed the probability of progression from liver cirrhosis to hepatocellular carcinoma under specific conditions: when AT3 levels were normal, the probability was 0.0749 (7.5%); when AT3 was low, it was 0.0709 (7.1%); and when AT3 was high, the probability increased to 0.0850 (8.5%). Regarding TC levels, the probability was 0.0763 (7.6%) when TC was normal, but rose significantly to 0.1309 (13.1%) when TC was elevated. The Netica node cumulative inference results showed that for a cirrhosis patient with hepatitis B, whose antithrombin III activity was at a low level and squamous cell carcinoma antigen, total cholesterol, and C-reactive protein were at high levels, the probability of progressing to hepatic malignancy was 52.1%. However, if antithrombin III activity, squamous cell carcinoma antigen, total cholesterol, and C-reactive protein were maintained at normal levels, the probability of developing hepatic malignancy would decrease to 7.5%, as shown in **Figure 6**.

### 3.6 Model evaluation

**Figure 7:**
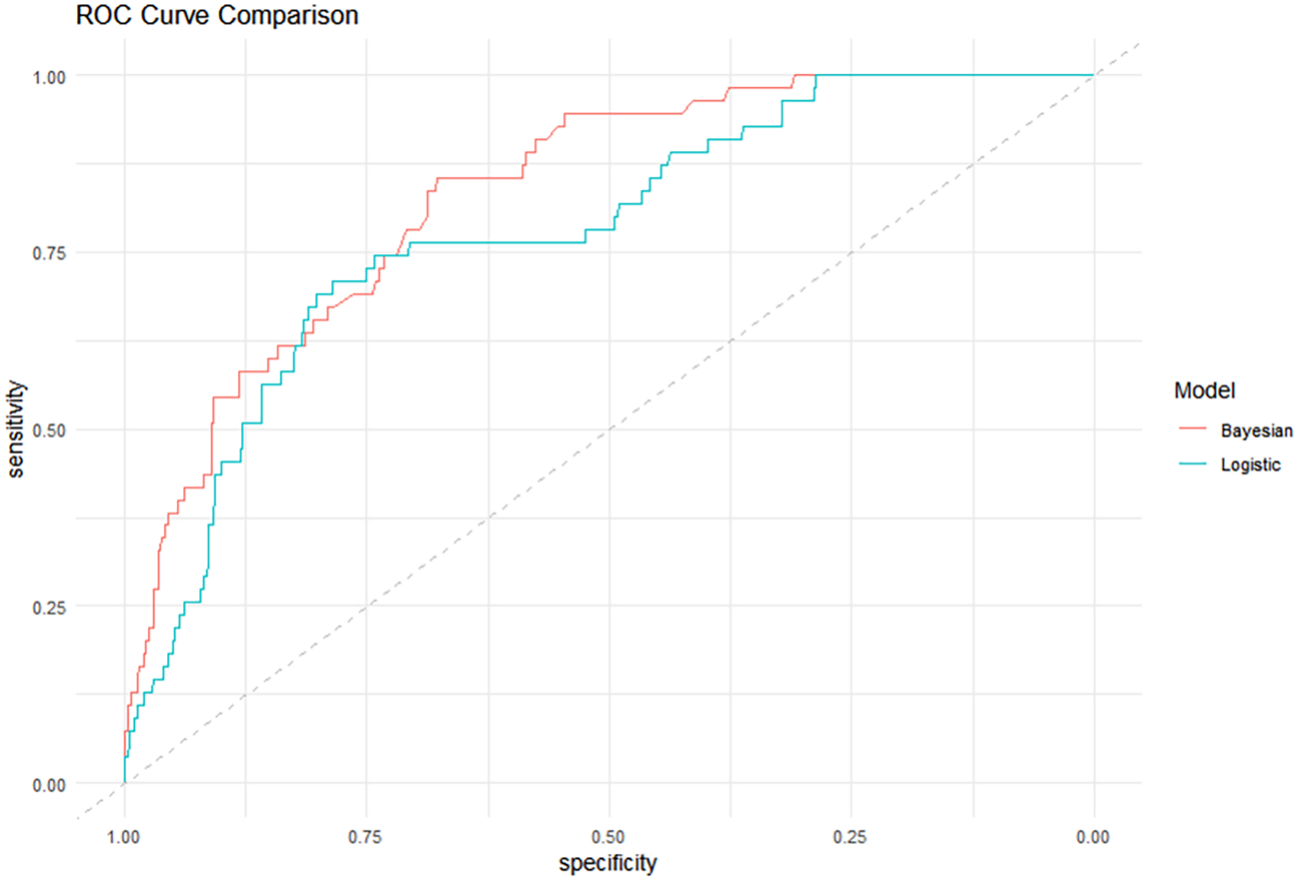
Comparison of the area under the ROC curve between the two models.

**Figure 8:**
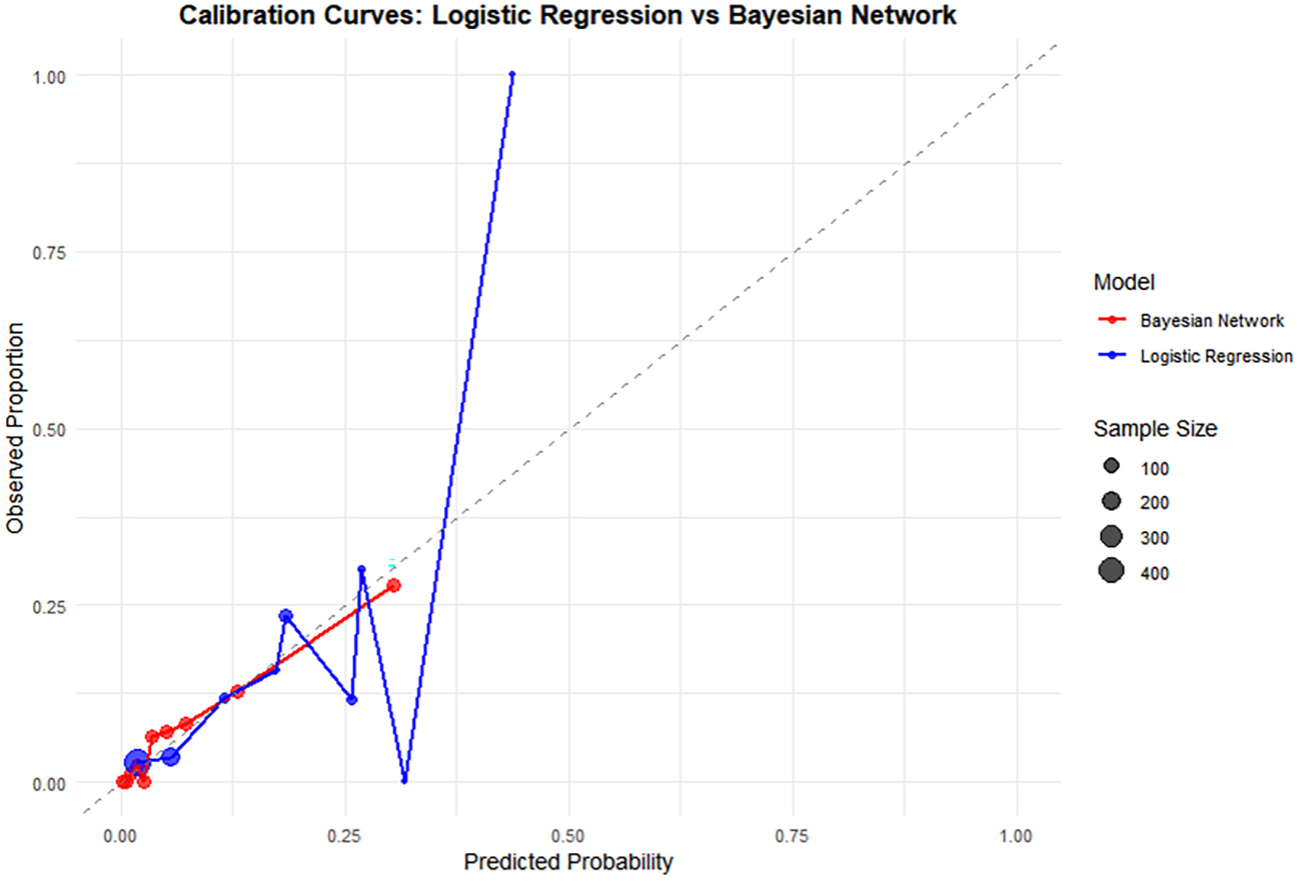
Comparison of the calibration curves between the two models.

The results showed that the performance of the Bayesian network model (AUC = 0.857, Brier Score = 0.052, Accuracy = 0.940, C-index (95% *CI*)=0.833(0.784–0.882), Sensitivity = 0.836, Specificity = 0.744) was superior to that of the logistic regression model (AUC = 0.780, Brier Score = 0.056, Accuracy = 0.934, C-index (95% *CI)* = 0.785 (0.726–0.843), Sensitivity = 0.727, Specificity = 0.766), as detailed in **Table 4** and **Figure 7**. The calibration curve results indicated that the predicted points of the Bayesian network model were evenly distributed around the diagonal line, suggesting good predictive performance (see **Figure 8**). Ten-fold cross-validation was performed, and the results showed that in nine out of the ten validation runs, the accuracy exceeded 0.9, with an average accuracy of 0.93 (see **Table 5**).

**Table 4:** Comparison of diagnostic performance between Bayesian network model and logistic regression model.

Model	Brier_Score	AUC	Accuracy	C-index(95%CI)	Sensitivity	Specificity
Bayesian Network	0.052	0.857	0.940	0.833(0.784-0.882)	0.836	0.744
Logistic Regression	0.056	0.780	0.934	0.785(0.726-0.843)	0.727	0.766

**Table 5:** Results of k-fold (10-fold) cross-validation.

k-fold	1	2	3	4	5	6	7	8	9	10	Mean
Accuracy	0.901	0.951	0.911	0.917	0.868	0.950	0.975	0.938	0.915	0.964	0.929

### 3.7 Model prediction threshold and risk stratification strategy for the Bayesian network model

**Figure 9:**
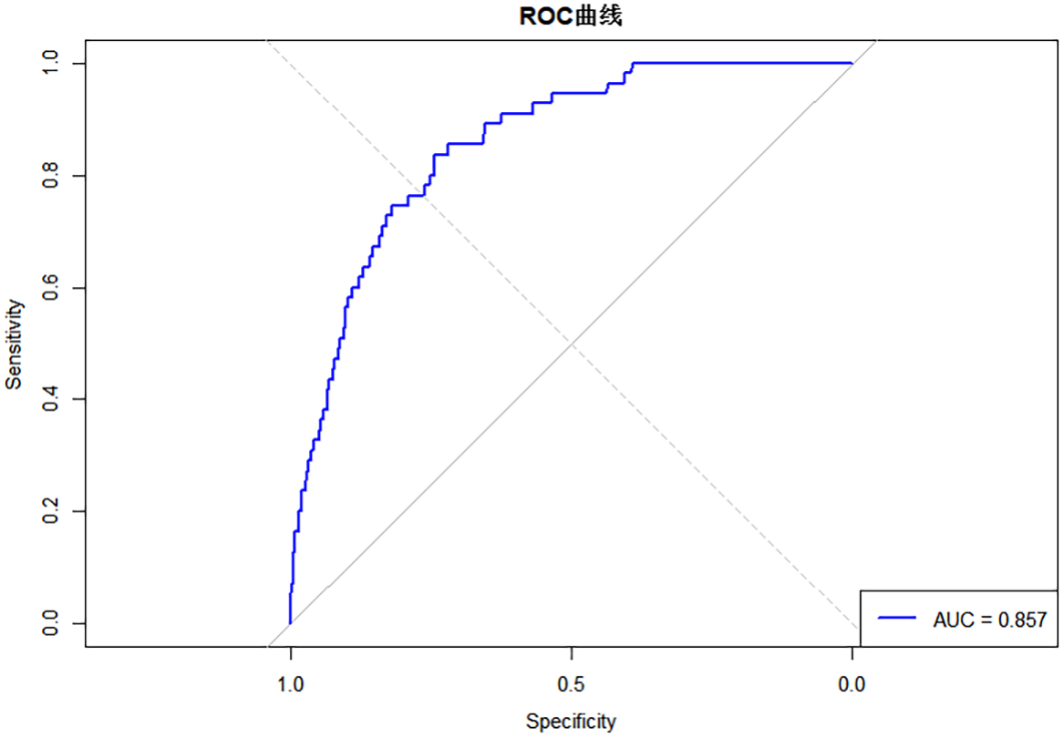
ROC curve of the Bayesian network model.

**Figure 10:**
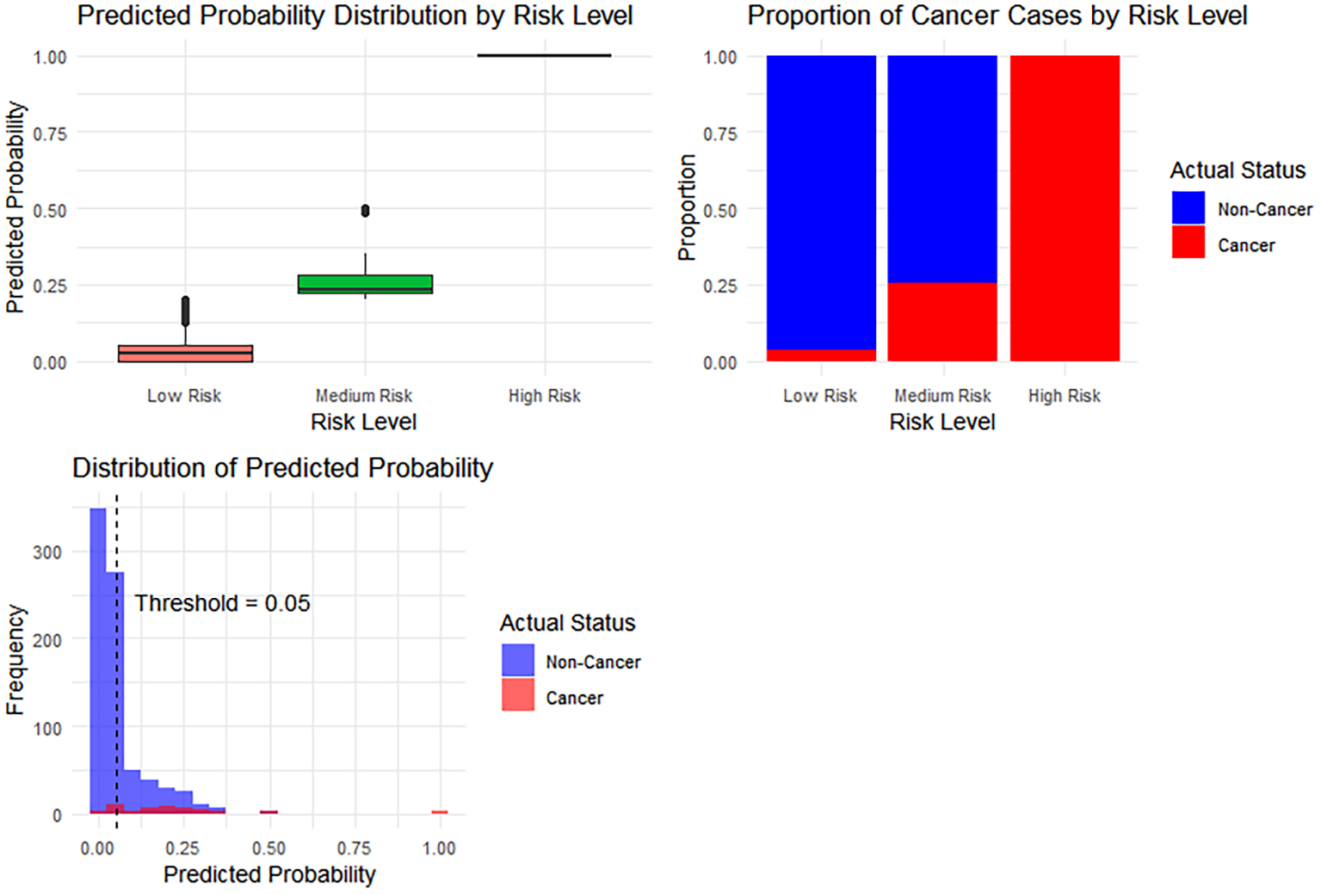
The relationship between risk strata and predicted probabilities, the relationship between risk strata and actual cancer status, and the predicted probability distribution.

Based on the predicted values, the ROC curve of the Bayesian network model was established (see **Figure 9**). The area under the curve (AUC) was 0.857. After balancing sensitivity and specificity, the optimal threshold was determined by maximizing the Youden index (0.052). A predicted probability > 0.052 indicates progression from liver cirrhosis to hepatic malignancy. Based on the ROC curve, threshold 1 was set at 0.2 and threshold 2 at 0.8 to establish a risk stratification system: low risk (predicted probability < 0.2), intermediate risk (0.2 ≤ predicted probability ≤ 0.8), and high risk (predicted probability > 0.8). Accordingly, 318 patients were classified as low risk, 42 as intermediate risk, and 1 as high risk (see **Table 6**). Monitoring frequency recommendations are as follows: low-risk patients: annual routine check-ups; intermediate-risk patients: regular monitoring every 6 months; high-risk patients: close monitoring every 3 months, with consideration for further diagnostic procedures. The relationships between risk strata and predicted probabilities, risk strata and actual cancer status, and the predicted probability distribution are shown in **Figure 10**.

**Table 6:** Risk stratification of the Bayesian network model.

Risk stratum	Sample size	Risk proportion
Low Risk	318	0.036
Intermediate risk	42	0.253
High Risk	1	1.000

## 4 Discussion

Hepatocellular carcinoma exhibits high mortality rates and poor prognosis, constituting a major disease affecting human health and quality of life. In China, over 80% of hepatocellular carcinoma cases develop from cirrhosis. Consequently, close monitoring of cirrhosis patients to prevent progression to hepatocellular carcinoma is paramount. This study enrolled 1,204 cirrhosis patients, among whom 76 progressed to hepatocellular carcinoma. Male cirrhosis patients exhibited a higher probability of developing hepatocellular carcinoma than female patients, a finding consistent with the gender incidence ratio observed in China, South Korea, and Japan (8). This may be related to the fact that men engage in unhealthy lifestyle habits such as smoking and excessive alcohol consumption more frequently than women (9, 10). A single-centre cohort study in the United States indicates that men are diagnosed with liver cancer at a later stage than women, presenting with larger tumours and a greater number of lesions. This may be attributed to men undergoing routine screening for liver cancer less frequently. Consequently, it is recommended that male patients with hepatitis, cirrhosis, and even those in good health undergo regular health check-ups to facilitate early detection and diagnosis of liver cancer. Prompt treatment is essential to enhance therapeutic outcomes. At the same time, men should place greater emphasis on lifestyle and dietary habits, refraining from smoking and excessive drinking, adopting a healthy diet, engaging in regular exercise, and enhancing their physical fitness.

This study found that patients with cirrhosis complicated by hepatitis B virus infection exhibited a higher risk of developing hepatocellular carcinoma compared to other patients. Furthermore, multivariate analysis revealed that the presence of hepatitis B virus infection constitutes a risk factor for the progression of cirrhosis to hepatocellular carcinoma. In China, the incidence of hepatitis B-related hepatocellular carcinoma remains persistently high, with studies predicting an increasing trend in this incidence over the forthcoming period (11, 12). Since the formulation of the Guidelines for the Prevention and Treatment of Chronic Hepatitis B in 2005, the prevalence of hepatitis B in China has shown a marked downward trend (13). Nevertheless, China remains one of the countries bearing the heaviest global burden of hepatitis B virus infection (14), posing an extremely serious threat to the lives and property of its people. Consequently, the prevention and control of hepatitis B cannot be delayed. Regular testing for hepatitis B antibodies and timely vaccination with the hepatitis B vaccine are effective methods for preventing infection with the hepatitis B virus. Rejecting drugs, avoiding the sharing of syringes, and maintaining healthy lifestyle and dietary habits to boost immunity can all effectively prevent hepatitis B infection.

Among hepatitis patients, the longer the duration of illness, the greater the likelihood of developing hepatocellular carcinoma. This finding is consistent with the results of a Chinese age-time cohort study on hepatocellular carcinoma (10). Rocio I.R. Macias et al. (15) propose that with increasing age and disease duration, factors such as genomic instability, telomere attrition, epigenetic alterations, impaired protein homeostasis, and mitochondrial dysfunction collectively promote the development of hepatocellular carcinoma. The liver possesses remarkable regenerative potential; however, over time, the accumulation of risk factors can lead to irreversible damage to liver function. Consequently, early detection, diagnosis, and treatment are essential measures for reducing the incidence of liver cancer. National and societal bodies should establish comprehensive liver cancer surveillance systems to promptly identify high-risk populations. Communities and hospitals must undertake public education responsibilities, providing health awareness campaigns to inform the public about the dangers of liver cancer and the necessity of timely diagnosis and treatment.

The relationship between serum cholesterol and the risk of hepatocellular carcinoma remains controversial. Findings from studies by Cai X et al. indicate a negative correlation between serum cholesterol levels and the incidence of hepatocellular carcinoma (16, 17), whereas other research suggests that elevated cholesterol levels may contribute to the development of this malignancy (18–20). Takuma Tsuchida et al. (21) observed in mouse studies that a high-cholesterol diet readily induces non-alcoholic fatty liver disease, subsequently leading to hepatocellular carcinoma. Further research indicates that dietary cholesterol drives hepatocellular carcinoma development by inducing alterations in the gut microbiota and metabolites of mice (19). This study found that patients with elevated cholesterol levels also exhibited a higher incidence of liver cancer. Analysis of influencing factors similarly indicated that elevated cholesterol constitutes a risk factor for the progression of cirrhosis to liver cancer, potentially linked to these patients’ high-cholesterol diets. Patients with cirrhosis require adequate nutrition to control disease progression, yet excessive cholesterol intake proves counterproductive. Given that high-fat, high-cholesterol foods constitute a significant proportion of the Xinjiang diet, promoting balanced nutrition and increasing physical activity are essential strategies for preventing the occurrence of liver cancer.

Antithrombin III, synthesised by the liver, vascular endothelial cells and megakaryocytes, is a protease inhibitor in plasma with anticoagulant properties. It can inhibit hepatitis viruses and reduce the likelihood of developing cirrhosis and hepatocellular carcinoma by modulating multiple host cell signalling factors associated with these conditions (22). It may also regulate the development of hepatocellular carcinoma by inhibiting thrombin (22), and can prevent the metastasis of cancer cells (23). This study has identified reduced antithrombin III activity as a risk factor for the progression of liver cirrhosis to hepatocellular carcinoma. This may be attributed to the more severe hepatic damage in cirrhotic patients, which diminishes their capacity to produce antithrombin III. Consequently, plasma antithrombin III levels decline, impairing the body’s ability to inhibit hepatocellular carcinoma development. Consequently, close monitoring of coagulation function is essential in daily life for cirrhosis patients. Regular medical examinations and adherence to prescribed medication are crucial for restoring antithrombin III activity. Concurrently, appropriate supplementation with iron- and folate-rich foods—such as animal blood, liver, and egg yolks—is advised to correct anaemia.

Bayesian networks can illustrate the relationship between various influencing factors and the progression of cirrhosis to hepatocellular carcinoma. The model indicates that, apart from phosphorus levels, BMI, thrombin time, and prothrombin time, other factors are associated with the development of hepatocellular carcinoma through direct or indirect pathways. Gender influences the type and duration of hepatitis, thereby contributing to HCC development. A 2019 study on the demographic characteristics of hepatitis B virus infection in China indicated that males are more susceptible to viral hepatitis than females (24). Patients with hepatitis B virus infection typically experience more severe disease and greater treatment challenges; the likelihood of developing HCC increases with disease progression.

This study further revealed that hepatitis type can influence free thyroxine levels, thereby affecting antithrombin III activity and ultimately contributing to hepatocellular carcinoma. Research indicates that nearly half of patients with viral hepatitis undergoing interferon therapy develop thyroid disorders, presenting as hypothyroidism (25). Prolonged hypothyroidism increases the risk of hepatocellular carcinoma (26), potentially through hypothyroidism-induced coagulation disorders leading to haemorrhage (27).

Squamous cell carcinoma antigen is a serine protease inhibitor present in normal squamous epithelium. It correlates with the invasion, metastasis, recurrence, and prognosis of squamous cell malignancies, serving as a crucial tumour marker reflecting the biological characteristics of squamous cell carcinoma. Research indicates elevated SCC-related antigen levels in the serum of hepatocellular carcinoma patients, whereas non-hepatocellular carcinoma patients exhibit lower serum SCC-related antigen concentrations (28). The Bayesian network model indicates a pathway from antithrombin III to SCC-A. However, no direct evidence currently exists demonstrating that antithrombin III activity directly influences SCC-A. This discrepancy may explain the elevated SCC-A levels observed in patients with abnormal antithrombin III activity in the present study.

Research indicates (29) that alterations in calcium ion concentration may influence the production and secretion of C-reactive protein. Under normal circumstances, hypocalcaemia (low blood calcium levels) may be associated with elevated C-reactive protein levels. This occurs because hypocalcaemia can stimulate the release of certain cytokines, thereby promoting C-reactive protein production. C-reactive protein is a non-specific inflammatory marker serving as an indicator of sensitivity and specificity in predicting tumour development (30). Existing research has identified an association between C-reactive protein and the occurrence of hepatocellular carcinoma, with higher C-reactive protein levels correlating with increased risk of liver cancer (31, 32). This aligns with the pathway demonstrated in this study’s model, wherein serum total calcium influences C-reactive protein, which in turn affects hepatocellular carcinoma development.

This study examined 1,204 patients with liver cirrhosis admitted to our hospital between 2019 and 2023, identifying 76 cases where cirrhosis progressed to hepatocellular carcinoma. Multivariate analysis revealed that hepatitis B infection, elevated total cholesterol levels, and reduced antithrombin III activity constitute risk factors for the development of malignant liver tumours in cirrhotic patients. Using the LASSO regression model for variable selection, fourteen variables most significantly associated with the progression from cirrhosis to malignant liver tumours were identified. These selected variables were incorporated into a Bayesian network. Combining literature evidence and decision tree model results, a risk prediction model for the progression from cirrhosis to hepatocellular carcinoma was constructed. In summary, gender, hepatitis type, disease duration, total calcium, C-reactive protein, total cholesterol, antithrombin III activity, free thyroxine, and carcinoembryonic antigen are closely associated with the progression of liver cirrhosis to hepatocellular carcinoma. This study has certain limitations. Firstly, the data utilised in this investigation was sourced from a single centre, namely the Xinjiang Uygur Autonomous Region People’s Hospital. Consequently, the distribution of study subjects may lack representativeness. Future research should involve collaboration with multiple hospitals across the region to enhance the representativeness of the study population. Secondly, all variables included in this study were extracted from patients’ hospital admission records and inpatient examination results, without considering variables such as lifestyle habits or dietary patterns. This may introduce bias into the model results. Questionnaire surveys or similar methods should be incorporated to broaden the scope of variables covered. Finally, the progression from cirrhosis to hepatocellular carcinoma is a protracted process. This study’s five-year observation period yielded a relatively small number of patients who developed hepatocellular carcinoma, potentially reducing the model’s accuracy. Consequently, subsequent research should combine retrospective and prospective studies to extend the observation period and minimise research error.

## Data Availability

The raw data supporting the conclusions of this article will be made available by the authors, without undue reservation.
